# Divergent Perceptions of Barriers to Diabetic Retinopathy Screening Among Patients and Care Providers, Los Angeles, California, 2014–2015

**DOI:** 10.5888/pcd13.160193

**Published:** 2016-10-06

**Authors:** Yang Lu, Lilian Serpas, Pauline Genter, Betty Anderson, David Campa, Eli Ipp

**Affiliations:** Author Affiliations: Yang Lu, Lilian Serpas, Pauline Genter, Betty Anderson, Harbor-UCLA Medical Center and Los Angeles Biomedical Research Institute, Torrance, California; David Campa, Los Angeles County Department of Health Services, Hubert H. Humphrey Comprehensive Health Center, Los Angeles, California.

## Abstract

**Introduction:**

Despite availability of screening for diabetic retinopathy, testing is underused by many low-income and racial/ethnic minority patients with diabetes. We examined perceived barriers to diabetic retinopathy screening among low-income patients and their health care providers and provider staffers.

**Methods:**

We collected survey data from 101 patients with diabetes and 44 providers and staffers at a safety-net clinic where annual diabetic retinopathy screening rates were low. Barriers specified in the survey were derived from the literature.

**Results:**

Patients surveyed (mean [standard deviation] age, 54.0 [7.7] y; 41% were male) were primarily Hispanics (70%) and African Americans (27%) of low socioeconomic status. Overall, 55% of patients received diabetic retinopathy screening in the previous year. Patients who could not explain why this screening is needed reported more barriers than patients who could (2.5 vs 1.4 barriers, *P* = .02). Fewer patients reported that they experienced barriers such as transportation (15%), language issues (15%), cultural beliefs or myths (4%), denial (8%), and fear (5%), which providers and staffers considered very or extremely important (all *P* < .001). Financial burdens (26%) and depression (22%) were most commonly reported by patients as barriers, yet providers and staffers did not rate these barriers as important, *P* < .001.

**Conclusion:**

Patients and health care providers had markedly divergent perceptions of barriers to diabetic retinopathy screening. Patients with poor understanding of the need for screening were more likely to report such barriers. These results suggest a need for active community engagement to find key elements for education programs and other interventions to increase rates of diabetic retinopathy screening, particularly among low-income, minority populations.

## Introduction

Diabetes is a major cause of vision impairment and blindness in the working-age population (18–65 y) in the United States and worldwide (1,2). More than a third of people with diabetes worldwide have some form of diabetic retinopathy (DR), and 10% have sight-threatening DR (3). The landmark Diabetes Control and Complications Trial determined intensive glycemic control in diabetes reduced the risk of development and progression of diabetic retinopathy by 34% (4). Conversely, increased hemoglobin A1C levels have been directly related to development of complications of diabetes, including DR (5).

Early detection and treatment of sight-threatening DR is best accomplished by routine screening as recommended by the American Diabetes Association and the American Academy of Ophthalmology (6,7). Yet, low use of DR screening is common, especially among low-income patients who are racial/ethnic minorities. In 2002, the DR screening rate for working-age adults in the United States was 56%. By 2009, this rate had increased to 59% for white patients with diabetes while the rate for minority patients with diabetes dropped to 49% (8). However, national data, such as data from the National Health and Nutrition Examination Survey and the National Health Interview Survey, indicate that people without a high school diploma or people at lower income levels have significantly higher rates of DR (9). In addition, low screening rates for DR in racial/ethnic minority patients demonstrate an association with a lack of understanding that diabetes can lead to complications such as DR (10). These findings indicate a greater need for increased DR screening and patient education among low-income minority patients (5,11).

Research suggests that multiple patient barriers to DR screening exist, including poor access to care, lack of time, out-of-pocket expenses, insufficient patient knowledge or awareness of DR, and lack of care coordination (12–17). However, few studies have examined whether patient barriers are recognized and understood by their health care providers, which is an important step toward improving patient screening compliance and diabetes outcomes, particularly among low-income and minority patients with low annual DR screening rates.

To fill this knowledge gap, we identified and compared perceived barriers to DR screening among low-income patients and their health care providers and provider staffers. To assess what barriers might be relevant without cost as a consideration, we studied patients in a setting where retinal screening was free of charge. Our findings may inform interventions to remove or ameliorate barriers to recommended diabetes care among vulnerable patient populations.

## Methods

A cross-sectional survey was designed to capture all potential barriers to retinopathy screening perceived by patients and by providers and their staffers. Specific barriers in the survey were identified and derived from the literature (12–17). Participants were also asked to report any additional barriers not listed in the survey. This anonymous patient survey was pilot-tested and was finalized on the basis of patient feedback to ensure all questions were culturally appropriate and were understood.

Patients were recruited from a large safety-net health center in South Los Angeles that serves predominantly low-income minority patients. The inclusion criteria for patients were that patients be aged 18 years or older and have a clinical diagnosis of diabetes. Data were collected anonymously from patients, providers, and staffers from December 2014 through January 2015. By using a convenience sample method, the survey team approached 110 patients waiting to be seen in the family medicine, internal medicine, or diabetes clinics and all 55 providers and provider staffers working in these 3 clinics, which comprise most of the health center. One hundred and one patients with diabetes and 44 providers and staffers completed the survey, resulting in a response rate of 92% and 80%, respectively. About two-thirds of the patient surveys were completed in Spanish and translated to English.

Patients were asked to rate any given barrier that “would delay or prevent you from getting your screening/test for diabetic eye disease” on a 5-point Likert scale with choice options of “strongly disagree,” “disagree,” “neither agree or disagree,” “agree,” and “strongly agree.” Health care providers and their staffers were asked to “check how important it is to address the following potential barriers for patients to receive retinal eye screening at [clinic]” on a 5-point Likert scale with choice options of “not at all important,” “slightly important,” “moderately important,” “very important,” and “extremely important.”

We used Pearson’s χ^2 ^to compare differences in perceived barriers to DR screening between patients and providers and staffers. If a patient agreed or strongly agreed with a barrier, we considered the patient as having identified that as a barrier. We examined whether the number or type of barriers reported differed by patient characteristics by using the 2-sided student *t* test. All statistical analysis was conducted in Stata, version 13 (StataCorp LP). The study was approved by the institutional review board of the Los Angeles Biomedical Research Institute.

## Results

Patients surveyed (mean [standard deviation] age, 54.0 [7.7] y; 41% were male) were primarily Hispanic (70%) and African American (27%) of low socioeconomic status (Table). Half reported an annual household income of less than $10,000, and 56% indicated they were not working. Six in 10 patients either were not married or did not live with a partner, and only one-third had a high school or general equivalency diploma or more education. Most had had diabetes for more than 5 years; duration of diabetes was more than 10 years in 40% of patients and from 6 to 9 years in 27%.

**Table:** Demogrpahic Characteristics of Participants (N = 101), Study of Barriers to Diabetic Retinopathy Screening in a Safety-Net Health Center in South Los Angeles, California, 2014–2015

Characteristic	n (%)^a^
**Age, y, mean (SD)**	54.0 (7.7)
**Male**	41 (40.6)
**Race/ethnicity**	
Hispanic	71 (70.3)
African American	27 (26.7)
Other	3 (2.0)
**Marital status**	
Single	37 (36.6)
Married or living with a partner	41 (40.6)
Widowed/divorced/separated	23 (22.8)
**Employment status**	
Employed/self-employed	44 (43.6)
Unemployed	29 (28.7)
Retired	6 (5.9)
Disabled	7 (6.9)
Other (housewife/student/no answer)	15 (14.8)
**Education**	
None	9 (8.9)
Some school, no diploma	58 (57.4)
High school or general equivalency diploma	22 (21.8)
Trade/technical/vocational training	2 (2.0)
Undergraduate degree or higher	9 (8.9)
**Annual household income, $**	
<10,000	50 (49.5)
10,000–50,000	40 (39.6)
No answer	11 (10.9)
**Diabetes duration, y**	
0–1	15 (14.9)
2–5	17 (16.8)
6–9	27 (26.7)
≥10	41 (40.6)
**Transportation to clinic**	
Drive oneself	34 (33.7)
Walk	7 (6.9)
Bus	37 (36.6)
Brought by someone	21 (20.8)

Abbreviations: SD, standard deviation.

a Values are number (percentage) unless otherwise indicated. Numbers may not add to 101 because of rounding.

Overall, 55% of patients reported that they had received DR screening in the previous year. This was despite the fact that 93% of patients were aware that diabetes can cause vision loss and blindness and despite 77% of patients having had their physician recommend they have DR screening. Lack of DR screening in the previous year was not associated with whether patients reported any barriers.

Further evaluation showed that 31% of patients reported no perceived barriers, 26% acknowledged 1 barrier, and 44% reported 2 or more barriers. Most commonly reported barriers were depression (22%) and financial problems (26%); 14% reported both. Language issues, lack of transportation, and lack of time were each reported as barriers by 15% of patients.

Employed patients were more likely to report lack of time to be a barrier than those not employed (23% vs 7%, *P* = .03). In general, women reported more barriers than men (1.9 vs 1.2, *P* = .06). Patients who could not adequately explain why DR screening is needed (n = 21) reported more barriers than patients who could (2.5 vs 1.4, *P* = .02).

**Figure 1:**
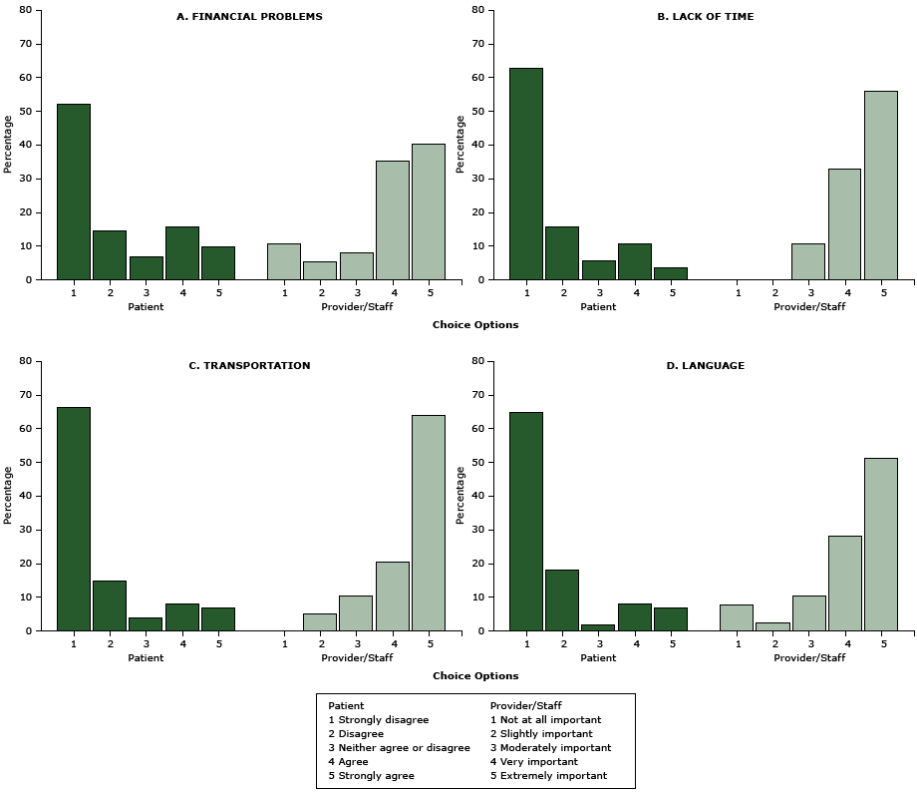
Perceived logistic and external barriers to diabetic retinopathy screening, Los Angeles, California, 2014–2015. Patients were asked to rate reasons that “would delay or prevent you from getting your screening/test for diabetic eye disease.” Health care providers and their staffers were asked to rate “how important it is to address the following potential barriers for patients to receive retinal eye screening” at the clinic. All *P* < .001. Barriers are ordered in descending order by how frequently they were identified by patients **Table:** 

**Figure 2:**
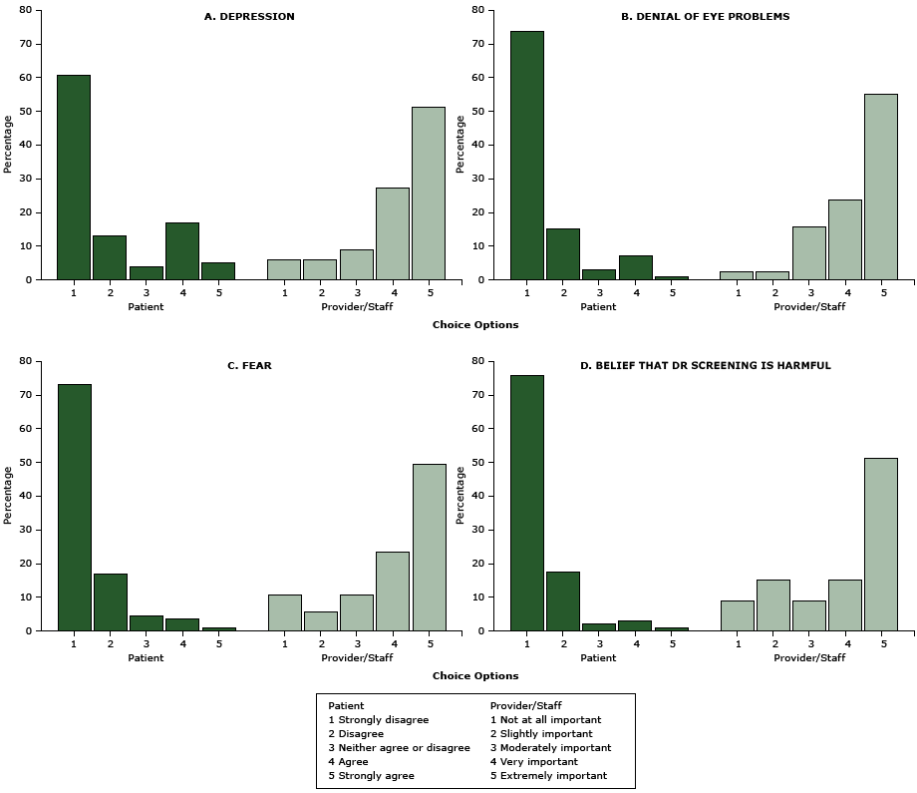
Perceived potential internal barriers to diabetic retinopathy screening, Los Angeles, California, 2014–2015. Patients were asked to rate reasons that “would delay or prevent you from getting your screening/test for diabetic eye disease.” Health care providers and their staffers were asked to rate “how important it is to address the following potential barriers for patients to receive retinal eye screening” at the clinic. All *P* < .001. Barriers are ordered in descending order by how frequently they were identified by patients. Abbreviation: DR, diabetic retinopathy. **Table:** 

The comparison of 8 barriers rated by patients versus those rated by providers and their staffers revealed markedly divergent perceptions between the 2 groups (Figure 1, Figure 2) (all *P* < .001). Only a small fraction of patients reported that they experienced barriers such as transportation (15%), language issues (15%), denial (8%), fear (5%), or cultural beliefs that DR screening is harmful (4%) that most providers and staffers thought to be very or extremely important (*P* < .001). In contrast, the barriers most commonly reported by patients — financial burdens (26%) and depression (22%) — were not considered as important as other barriers by providers and staffers (*P* < .001). Furthermore, even though 93% of patients were aware diabetes can cause vision loss and blindness, in contrast, 70% of providers and staffers rated lack of such awareness as “extremely important.” Similarly, 77% of patients were aware of a DR screening program in the clinic, whereas more than half of providers and staffers rated lack of such awareness as “extremely important” (*P* < .001).

Choice Option	Patient Perception, %	Provider/Staff Perception, %
**Financial problems**		
1	Strongly disagree, 52.5%	Not at all important, 10.8%
2	Disagree, 14.9%	Slightly important, 5.4%
3	Neither agree or disagree, 6.9%	Moderately important, 8.1%
4	Agree, 15.8%	Very important, 35.1%
5	Strongly agree, 9.9%	Extremely important, 40.5%
**Lack of time**		
1	Strongly disagree, 63.0%	Not at all important, 0.0%
2	Disagree, 16.0%	Slightly important, 0.0%
3	Neither agree or disagree, 6.0%	Moderately important, 11.1%
4	Agree, 11.0%	Very important, 33.3%
5	Strongly agree, 4.0%	Extremely important, 55.6%
**Transportation**		
1	Strongly disagree, 66.3%	Not at all important, 0.0%
2	Disagree, 14.9%	Slightly important, 5.1%
3	Neither agree or disagree, 4.0%	Moderately important, 10.3%
4	Agree, 7.9%	Very important, 0.5%
5	Strongly agree, 6.9%	Extremely important, 64.1%
**Language**		
1	Strongly disagree, 65.0%	Not at all important, 7.7%
2	Disagree, 18.0%	Slightly important, 2.6%
3	Neither agree or disagree, 2.0%	Moderately important, 10.3%
4	Agree, 8.0%	Very important, 8.2%
5	Strongly agree, 7.0%	Extremely important, 51.3%

Choice Option	Patient Perception	Provider/Staff Perception
**Depression**		
1	Strongly disagree, 61.0%	Not at all important, 6.1%
2	Disagree, 13.0%	Slightly important, 6.1%
3	Neither agree or disagree, 4.0%	Moderately important, 9.1%
4	Agree, 17.0%	Very important, 27.3%
5	Strongly agree, 5.0%	Extremely important, 51.5%
**Denial of eye problems**		
1	Strongly disagree, 74.0%	Not at all important, 2.6%
2	Disagree, 15.0%	Slightly important, 2.6%
3	Neither agree or disagree, 3.0%	Moderately important, 15.8%
4	Agree, 7.0%	Very important, 23.7%
5	Strongly agree, 1.0%	Extremely important, 55.3%
**Fear**		
1	Strongly disagree, 73.3%	Not at all important, 10.5%
2	Disagree, 16.8%	Slightly important, 5.3%
3	Neither agree or disagree, 5.0%	Moderately important, 10.5%
4	Agree, 4.0%	Very important, 23.7%
5	1 Strongly agree,.0%	Extremely important, 50.0%
**Belief that DR screening is harmful**		
1	Strongly disagree, 76.3%	Not at all important, 9.1%
2	Disagree, 17.5%	Slightly important, 15.2%
3	Neither agree or disagree, 2.1%	Moderately important, 9.1%
4	Agree, 3.1%	Very important, 15.2%
5	Strongly agree, 1.0%	Extremely important, 51.5%

## Discussion

Although most patients in this study (93%) understood that diabetes can lead to eye disease and three-quarters received a physician recommendation for DR screening, the annual screening rate was only 55%, comparable to previously reported rates for this population (8,18). This low screening rate confirms the suitability of this group of patients to represent the low-income, minority patient population we were studying, many of whom did not use diabetes retinal screening despite having knowledge of its importance. The study goal was not only to understand patient barriers but also to assess provider understanding of their patients’ barriers. Recent meta-analysis showed interventions involving shared decision making between patients and physicians might benefit disadvantaged patients to a greater extent than patients of higher socioeconomic status, particularly if these interventions were adequately tailored to the specific needs of the patient groups (19). These low screening rates may be partly due to providers’ poor understanding of how patients perceive barriers to DR screening, as demonstrated in the study.

Our findings show that provider and staffer perceptions of barriers to DR screening were remarkably different from those of their patients. Given that 77% of patients in the study had received a physician’s recommendation for DR screening, this vast difference suggests a lack of high-quality patient–provider communication regarding patient barriers to DR screening during the referral process. In particular, financial burden and depression were most often reported by patients but deemed less important by providers and staffers than other barriers. There was no direct out-of-pocket expense for DR screening at the safety-net health center where the study was conducted. This may explain why providers and staffers did not think financial burden was a barrier. However, 26% of patients perceived it so, suggesting that hidden costs may be high. Sloan et al presented an economic framework for nonmonetary costs of DR screening, such as perceived rate of vision change (very slow, if at all), the estimated functional vision loss from lack of timely action, and perceived effectiveness of any intervention (20). These factors may have influenced patients’ perceptions. Other considerations may also have been opportunity costs, such as long waits at clinics and duration of the screening procedure, which disproportionately affect minority patients, are potentially correctible, and warrant further investigation (21).

Financial loss from time off work likely also affects patients who are employed, particularly those without a flexible work schedule. Depression was the other common barrier reported by patients. Providers and staffers did not perceive depression as important, but the barrier is plausible given its high prevalence in patients with diabetes (22–24). In addition, depression has been associated with increased severity of diabetes complications, including retinal abnormalities. Even after accounting for blood pressure, smoking history, and other factors, patients with diabetes and depression have wider retinal arterioles than patients with diabetes without depression (25). This finding is a possible early indicator of microvascular disease that precedes typical findings of diabetic retinopathy, which suggests a more immediate need for timely DR screening for patients with diabetes and depression. Furthermore, 60% of patients in our study did not have a spouse or live-in partner. For them, depression may be a more critical barrier because of inadequate family support for diabetes care. Low levels of social support have been implicated in low screening rates for cardiovascular conditions (26). A similar phenomenon may be occurring in patients with diabetes.

Lack of time is another barrier of interest, especially affecting patients who were employed. Given low levels of household income reported in the survey, employed patients in our study likely held nonmanagerial jobs with inflexible time schedules, making medical appointments difficult to arrange. To facilitate the process, DR screening was provided in the primary care setting where the study took place. But it generally required a separate referral and appointment, which may make it challenging for these patients to use the service.

The survey also revealed a connection between lack of understanding of the need for DR screening and the number of reported barriers in the patient group. This association between the ability to understand the need for a procedure and perception of barriers suggests that targeted education might be helpful to increase DR screening rates and lower perception of barriers. The goal is increased patient understanding of DR screening and therefore its benefit. Perceived benefit is associated with greater likelihood of compliance with procedures (27), perhaps by influencing the risk–benefit calculation. Along with perceived benefit, perceived priority of DR screening is probably also a factor. A recent study incorporated the Health Belief Model construct into a large patient survey and found that patient-held health beliefs were strong predictors of their adherence to DR screening, including whether the patient thought a DR screening was a “top priority” (17). Coupled with a lack of knowledge among minority patients as to whether inadequate diabetes control leads to complications such as DR (10), disadvantaged patients may have false perceptions regarding the importance or priority of DR screening. Education that aims to improve diabetes knowledge and self-management skills among patients is crucial in helping them understand the importance of adequate diabetes care, including prevention of diabetes-related complications, such as timely DR screening (5,11,28).

The 31% of patients who reported no barriers did not show higher rates of DR screening, suggesting that patients might be unaware of all the barriers they face. It is also possible some barriers were not identified by our literature-based survey. Most providers and staffers rated all barriers as important. This suggests that the barriers identified in the literature reflect the perceptions of providers more than that those of patients, causing potential provider bias in surveys. Further supporting the notion that DR screening surveys may be inadequate in capturing patient perceptions is the large discrepancy between DR screening rates of African American and Hispanic patients, which could not be explained by any difference in reporting of barriers (29).

A limitation of this study is that in an effort to keep the survey succinct we did not ascertain whether the patients had had type 1 or type 2 diabetes. Because of the sample size, there would not have been enough power to differentiate responses between patients with type 1 and type 2 diabetes. However, it is likely that 95% of the surveyed patients had type 2 diabetes, given its estimated prevalence in minority populations. Another limitation is that the survey data were collected in a clinic; thus, we were unable to capture the perspectives of patients who may not regularly attend clinic visits. Qualitative or quantitative differences may exist in perception of barriers in patients lost to follow up or not attending clinic visits for other reasons. Those patients might perceive different types of barriers, or the same types of barriers, to a greater extent than the patients surveyed. Future research should engage patient communities to better capture perceived barriers and help understand what could improve DR screening rates among low-income minority patients, taking into account their unique medical needs.

Many barriers to DR screening acknowledged by low-income minority patients were anticipated, but the striking divergence between patient perception and provider and staffer perception of their importance was not. The lack of agreement between patients’ perceived barriers and provider and staffers’ understanding of those same barriers indicates need for more effective patient–provider communication and for patient feedback on health care delivery systems. Heightened awareness of depression and its impact on DR screening rates among low-income minority patients with diabetes is also key to improving their use of DR screening. In addition, a lack of knowledge of the importance of DR screening might prevent patients from identifying barriers that prevent them from adhering to secondary diabetes prevention, such as DR prevention and detection. Thus, patient education targeting diabetes knowledge and self-management is also crucial. Overall, these findings highlight the importance of engaging both minority patient communities and their health care providers in efforts to increase DR screening rates and prevent blindness.
